# The association between the transition to parenthood and risk for nonfatal suicide attempt in a Swedish population-based sample

**DOI:** 10.1017/S0033291726103262

**Published:** 2026-01-14

**Authors:** Mallory Stephenson, Henrik Ohlsson, Kenneth S. Kendler, Jan Sundquist, Alexis C. Edwards, Kristina Sundquist, Severine Lannoy

**Affiliations:** 1 Virginia Commonwealth University, USA; 2 Lund University: Lunds Universitet, Sweden

**Keywords:** father, mother, parenthood, suicide attempt

## Abstract

**Background:**

Parenthood is consistently identified as a protective factor for suicidal behavior. However, it remains unclear whether this relationship varies as a function of sex, age, time since birth, number of children, and other risk/protective factors.

**Methods:**

We used Cox proportional hazards models to describe the relationship between the birth of up to four children and suicide attempt (SA) risk in Swedish individuals born between 1960 and 1980. Models were stratified by sex and controlled for a range of covariates. We tested whether the relationship between parenthood and SA risk varies based on age at first birth and explored whether SA risk differed based on education, genetic liability, cohabitation with one’s co-parent, and geographic proximity to one’s mother (the child’s grandmother).

**Results:**

The first year following childbirth was associated with reduced SA risk in mothers (hazard ratios [HRs] = 0.34–0.64) and fathers (HRs = 0.60–0.86). However, later time periods following the birth of one’s third and fourth children were associated with elevated risk (HRs = 1.02–1.26). Moreover, age at first birth moderated the association between parenthood and SA: individuals who became parents at age 15 exhibited increased risk for SA (HRs = 2.81–5.30), while individuals with an older age at first birth (30+ years) experienced a reduction in risk (HRs = 0.31–0.92). The effect of parenthood also varied based on cohabitation and proximity to one’s mother.

**Conclusions:**

These findings underscore the complexity of the relationship between parenthood and SA, indicating that there are some subgroups for whom the transition to parenthood is not protective. Clinical outreach may be warranted as a preventative measure.

The relationship between parenthood and reduced suicide risk has long been of interest in the suicide literature. The first data were presented by Durkheim (2012), who concluded that men who were married with children had the lowest risk of suicide. A reanalysis of these data suggested that the inverse association between family life and suicide was primarily driven by parenthood rather than marital status (Veevers, 1973); as a result, Veevers (1973) proposed that the experience of raising children may facilitate the development of psychological stability and emotional maturity, contributing to decreased suicide risk. Over the subsequent 50 years, a number of large population-based studies have provided further empirical support for parenthood as a protective factor (Andrés, Collings, & Qin, 2010; Clark & Fawcett, 1994; Dehara et al., 2021; Høyer & Lund, 1993; Qin & Mortensen, 2003; Sörberg Wallin et al., 2022), with a linear reduction in suicide risk according to the number of children (Høyer & Lund, 1993).

The overall protective effect of parenthood is in line with leading theories of suicide (Klonsky & May, 2015; Van Orden et al., 2010), supporting the notion that parenthood may offer a unique sense of connectedness and belongingness, thereby reducing risk for suicidal thoughts and behaviors. Nevertheless, observational findings also indicate a high prevalence of maternal anxiety and depression that extends beyond the first postpartum year, particularly in marginalized subgroups (Boden, Fergusson, & John Horwood, 2008; Hunter, Chiew, McDonald, & Adhikari, 2024; Pearson et al., 2019). This effect is also observed in fathers (Fogarty et al., 2024; Smythe, Petersen, & Schartau, 2022), suggesting that some aspects of the parenthood experience may increase stress and suicide risk for some individuals and/or during specific periods of time.

To provide a comprehensive understanding of the relationship between parenthood and risk for suicidal behavior, we leveraged data from Swedish population-based registries to address six major questions. First, does the protective effect of parenthood extend to nonfatal suicidal behavior? To our knowledge, prior studies have only considered suicide death as an outcome (e.g. Qin & Mortensen, 2003; Sörberg Wallin et al., 2022), though the etiologies of suicide attempt (SA) and death are partially distinct (Beautrais, 2001; Edwards et al., 2021), and nonfatal SAs also constitute a serious public health concern (Prabhakar & Rice, 2023; World Health Organization, 2021). Second, does the relationship between parenthood and risk for SA persist after accounting for key confounders, including parental education, internalizing and externalizing disorders, marital status, and genetic liability?

Third, are there differences in the effect of parenthood on SA according to the number of children and their ages? There is some evidence to suggest that the protective effect of parenthood is stronger for parents of younger children (Qin & Mortensen, 2003; Sörberg Wallin et al., 2022), but relatively few studies have empirically tested this question. Fourth, does the direction or magnitude of the parenthood-SA association differ based on the age at which individuals become first-time parents? In the depression literature, younger ages at first birth are associated with increased risk of depression, whereas the transition to parenthood at an older age (~age 30) appears to be protective (Guo et al., 2024; Mirowsky & Ross, 2002; Pearson et al., 2019).

Fifth, is the potential protective effect of parenthood on SA risk related to structural social support? Because the current study relied on nationwide registries, cohabitation (i.e. whether individuals lived in the same residence as their co-parent or separately) and geographical proximity to parents were used as proxies for available support. Sixth, does parenthood interact with genetic liability and/or socioeconomic status (indexed by family education) to predict SA risk? Here, we capitalize on prior studies showing associations between genetic liability and/or family education and SA (Docherty et al., 2023; Edwards et al., 2021; Kendler, Ohlsson, Sundquist, Sundquist, & Edwards, 2020; Lannoy et al., 2022; Rosoff, Kaminsky, McIntosh, Davey Smith, & Lohoff, 2020) to investigate whether those who are at elevated risk of SA similarly benefit from the experience of parenthood. Addressing these questions will provide valuable insight into the contexts in which parenthood is protective against suicidal behavior and the contexts in which parents may be at risk. These insights can, in turn, inform the development of targeted prevention and intervention efforts.

## Method

### Sample

Data were drawn from Swedish population-based registries with national coverage. Records are linked using the Swedish 10-digit personal identification numbers assigned at birth, which are replaced with serial numbers to maintain confidentiality. The current analyses included all individuals born in Sweden between 1960 and 1980 who were residing in Sweden at age 15 (*N* = 2,156,741). Individuals were followed from age 15 until first registration for SA, death, emigration from Sweden, or December 31, 2018, whichever came first. The study was approved by the Regional Ethical Review Board of Lund University. Subject consent for nationwide registers is not needed.

### Measures

Nonfatal SAs were identified through the Swedish National Patient Register and the Primary Care Database using ICD-10 codes X60–X84 and Y10–Y34, and ICD-9 codes E950–E959 and E980–E989. Information on parenthood was obtained from the Swedish Multigenerational Register, which provided data on each individual’s first four biological children. In the case of twins (*N* = 10,233), one was randomly designated as child one and the other as child two.

#### Covariates

All models were controlled for birth year. Fully adjusted models additionally accounted for one’s parents’ years of education (standardized by sex and birth year), externalizing and internalizing psychiatric disorders, marital status, and family genetic risk scores for SA (FGRS_SA_). Externalizing and internalizing disorders were coded as binary time-varying variables. For externalizing disorders, individuals were coded as 0 until their first registration for alcohol use disorder, drug use disorder, or criminal behavior, after which they were coded as 1. For internalizing disorders, individuals were coded as 0 until their first registration for an anxiety or depressive disorder, after which they were coded as 1. Marital status was dummy-coded as married, divorced, or never married (reference group). FGRS_SA_ was calculated based on registrations for SA among first- to fifth-degree relatives. Scores accounted for the individual’s genetic relatedness to each relative (e.g. 0.5 for parents and full siblings and 0.125 for first cousins), the relative’s age at first registration with SA (when applicable), years of cohabitation, and differences in the number of relatives for whom information is available (Kendler, Ohlsson, Sundquist, & Sundquist, 2021). Further details on these variables are provided in the Supporting Information.

#### Indices of structural social support

Subsequent analyses evaluated whether the association between parenthood and SA risk may vary as a function of cohabitation with one’s co-parent during the first postpartum year and geographic proximity to one’s mother. Additional details are provided in the Supporting Information.

### Statistical methods

#### Preliminary analyses

First, we used Cox proportional hazards models to evaluate the crude association between the birth of one’s first child and subsequent SA risk, with separate analyses conducted for females and males. Parenthood status was treated as a time-varying predictor, such that an individual was considered unexposed until the birth of their first child. Separate hazard ratios (HRs) were estimated in 1-year intervals for up to 10 years following the birth of the child. Parameters that were not significantly different across years were equated, and model fit was assessed using the Akaike Information Criterion (AIC). For females, the parameters for years 5–10 could be equated. Therefore, for all subsequent analyses, we present HRs for six distinct time intervals: 0–1, 1–2, 2–3, 3–4, 4–5, and 5–10 years after childbirth. For males, parameters for years 3–10 could be equated, resulting in four time intervals in subsequent models (i.e. 0–1, 1–2, 2–3, and 3–10 years).

#### Overall models

In Model A, we examined risk for SA following the birth of an individual’s first child, adjusting for year of birth. Model B extended this analysis by incorporating the birth of up to four children. Model C considered the birth of up to four children and included additional covariates: mean years of parental education, externalizing and internalizing psychiatric disorders, marital status, and FGRS_SA_. Externalizing disorders, internalizing disorders, and marital status were included as time-varying covariates. Separate analyses were conducted for females and males.

#### Considering time-dependent effects for age at first birth

Next, we examined whether the association between the birth of one’s first child and risk for SA varied based on parental age at birth. To do this, we included an interaction term between the parameters of interest and the logarithm of age. We present the predicted HRs at 5 different ages (15, 20, 25, 30, and 35 years).

#### Evaluating moderation by FGRS_SA_ and parental education

We then tested whether the relationship between first-time parenthood and risk for SA was moderated by FGRS_SA_ or parental education. These models included an interaction term between time since birth and the moderator of interest. Each moderator was tested in a separate model.

#### Investigating the roles of structural social support

Finally, to explore the possibility that the magnitude of the association between the transition to parenthood and risk for SA may vary according to indices of structural social support, we tested two additional models. First, we allowed the effect of parenthood to vary as a function of cohabitation. We investigated this effect during the first postpartum year and divided the effect into two parts: one for cohabitation with co-parent and one for residing alone. In a separate model, we applied this approach with geographic proximity to one’s mother (the child’s grandmother). Here, we divided the effect into three parts: residing in the same demografiska statistikområden (DESO) area, the same municipality, or a different municipality. (DESO divides Sweden into 5,984 areas, with a target population of 1,500 individuals per area.)

All statistical analyses were performed using SAS 9.4.

## Results

Descriptive statistics are presented in Table 1. Approximately 80% of females and 72% of males had at least one child during the study period. The average age at first birth was 27.8 and 30.1 years in females and males, respectively.

**Table 1.** Descriptive statistics for the primary study variables

	N (%)/M (SD)	
	Females	Males
N	1,049,142	1,107,599
Suicide attempt	42,048 (4.0%)	42,327 (3.8%)
Age at first suicide attempt	30.8 (10.9)	33.5 (10.2)
At least 1 child	840,574 (80.1%)	800,506 (72.3%)
At least 2 children	707,938 (67.5%)	648,312 (58.5%)
At least 3 children	253,644 (24.2%)	226,213 (20.4%)
At least 4 children	60,448 (5.8%)	55,066 (5.0%)
Age at first birth	27.8 (5.2)	30.1 (5.4)
Parental education (years)	10.9 (2.8)	10.9 (2.8)
Externalizing disorder	101,961 (9.7%)	271,859 (24.5%)
Internalizing disorder	289,462 (27.6%)	180,442 (16.3%)
Marital status		
Married	649.548 (61.9%)	615,131 (55.5%)
Divorced	190,382 (18.2%)	163,500 (14.8%)
Never married	399,594 (38.1%)	492,468 (44.5%)
FGRS_SA_	0.0 (0.9)	0.0 (0.8)
Residential information 0–1 year after birth of first child		
Resides with co-parent	781,366 (93.0%)	758,452 (94.7%)
Resides in the same DESO as mother	104,790 (12.5%)	116,428 (14.5%)
Resides in the same municipality as mother	285,130 (33.9%)	274,085 (34.2%)

*Note:* Individuals with a registration for alcohol use disorder, drug use disorder, or criminal behavior were coded positively for externalizing disorders. Individuals with a registration for any depressive or anxiety disorder were coded positively for internalizing disorders. DESO, demografiska statistikområden; FGRS_SA_, family genetic risk score for suicide attempt; M, mean; SD, standard deviation.

Below, we review results from models of the association between parenthood and SA risk. We primarily focus on statistically significant effects due to space limitations.

### Overall models in mothers

We used Cox proportional hazards models to evaluate the relationship between parenthood and risk for SA. As shown in Table 2, the first 10 years following the transition to first-time parenthood were consistently associated with decreased risk for SA in mothers (Model A; HRs = 0.33–0.76). When we further accounted for the birth of (up to) four children (Model B), the first 4 years after the birth of one’s first child were still associated with lower risk for SA (HRs = 0.33–0.89). Decreased SA risk was also observed for 10 years after the birth of one’s second child (HRs = 0.36–0.77) and for the first 2 years after the birth of one’s third child (HRs = 0.64–0.81). Conversely, later time periods following the birth of one’s third and fourth children were related to *increased* SA risk in some cases (HRs = 1.09–1.77).

**Table 2.** Cox models of the relationship between the transition to parenthood and risk for suicide attempt in mothers

	HR [95% CI]		
	Model A	Model B	Model C
Time from birth of Child 1			
0–1 years	**0.33 [0.30, 0.36]**	**0.33 [0.30, 0.36]**	**0.34 [0.31, 0.38]**
1–2 years	**0.43 [0.40, 0.47]**	**0.45 [0.41, 0.49]**	**0.48 [0.44, 0.52]**
2–3 years	**0.53 [0.49, 0.57]**	**0.67 [0.61, 0.72]**	**0.69 [0.63, 0.75]**
3–4 years	**0.63 [0.58, 0.68]**	**0.89 [0.82, 0.97]**	**0.87 [0.81, 0.95]**
4–5 years	**0.72 [0.67, 0.77]**	1.03 [0.94, 1.12]	0.99 [0.92, 1.07]
5–10 years	**0.76 [0.73, 0.79]**	0.99 [0.94, 1.04]	0.96 [0.92, 1.00]
Time from birth of Child 2			
0–1 years		**0.36 [0.32, 0.41]**	**0.41 [0.36, 0.46]**
1–2 years		**0.56 [0.51, 0.62]**	**0.66 [0.60, 0.73]**
2–3 years		**0.64 [0.58, 0.70]**	**0.78 [0.71, 0.85]**
3–4 years		**0.65 [0.59, 0.71]**	**0.78 [0.72, 0.86]**
4–5 years		**0.72 [0.66, 0.79]**	**0.87 [0.80, 0.95]**
5–10 years		**0.77 [0.73, 0.80]**	**0.91 [0.87, 0.95]**
Time from birth of Child 3			
0–1 years		**0.64 [0.54, 0.75]**	**0.54 [0.46, 0.64]**
1–2 years		**0.81 [0.70, 0.94]**	**0.71 [0.62, 0.82]**
2–3 years		1.03 [0.90, 1.16]	0.92 [0.81, 1.05]
3–4 years		1.12 [0.99, 1.26]	1.02 [0.90, 1.15]
4–5 years		**1.33 [1.19, 1.49]**	**1.22 [1.10, 1.37]**
5–10 years		**1.09 [1.03, 1.15]**	**1.05 [0.99, 1.11]**
Time from birth of Child 4			
0–1 years		0.91 [0.70, 1.18]	**0.64 [0.49, 0.82]**
1–2 years		1.14 [0.91, 1.42]	0.80 [0.64, 1.00]
2–3 years		**1.30 [1.06, 1.60]**	0.91 [0.74, 1.12]
3–4 years		**1.57 [1.30, 1.90]**	1.07 [0.88, 1.29]
4–5 years		**1.67 [1.39, 2.01]**	1.12 [0.93, 1.35]
5–10 years		**1.77 [1.62, 1.93]**	**1.13 [1.04, 1.24]**
Covariates			
Year of birth	**1.02 [1.02, 1.02]**	**1.02 [1.02, 1.02]**	**1.02 [1.02, 1.02]**
Parental education			**0.91 [0.90, 0.92]**
Externalizing disorder			**5.84 [5.70, 5.98]**
Internalizing disorder			**6.49 [6.28, 6.71]**
Married			**0.90 [0.87, 0.93]**
Divorced			**1.65 [1.59, 1.72]**
FGRS_SA_			**1.27 [1.26, 1.28]**

*Note:* Model A focused on first-time parenthood and was adjusted for year of birth only. Model B further considered the birth of (up to) four children. In Model C, parental education, externalizing and internalizing disorders, marital status, and family genetic risk scores for suicide attempt were included as additional covariates. Statistically significant parameter estimates are shown in bold font. *Abbreviations:* CI, confidence interval; FGRS_SA_, family genetic risk score for suicide attempt; HR, hazard ratio.

Parameter estimates did not shift substantively when parental education, externalizing and internalizing disorders, marital status, and FGRS_SA_ were included as additional covariates (Model C), except that the associations between the birth of one’s fourth child and elevated SA risk were attenuated and became non-significant in all but one case. In addition, the first year after the birth of one’s fourth child was related to reduced SA risk in this fully adjusted model.

### Overall models in fathers

Parameter estimates for the association between parenthood and risk for SA in fathers are shown in Table 3. The birth of one’s first and second children were associated with lower risk for SA across Models A, B, and C (HRs = 0.55–0.94). Later time periods following the birth of one’s third and fourth children were associated with increased risk in some, but not all, cases (HRs = 1.07–1.73).

**Table 3.** Cox models of the relationship between the transition to parenthood and risk for suicide attempt in fathers

	HR [95% CI]		
	Model A	Model B	Model C
Time from birth of Child 1			
0–1 years	**0.55 [0.51, 0.60]**	**0.55 [0.51, 0.60]**	**0.60 [0.55, 0.65]**
1–2 years	**0.73 [0.68, 0.79]**	**0.75 [0.69, 0.80]**	**0.82 [0.76, 0.88]**
2–3 years	**0.75 [0.69, 0.80]**	**0.85 [0.79, 0.92]**	**0.90 [0.84, 0.97]**
3–10 years	**0.84 [0.82, 0.87]**	1.02 [0.98, 1.06]	1.01 [0.98, 1.05]
Time from birth of Child 2			
0–1 years		**0.61 [0.55, 0.67]**	**0.70 [0.64, 0.77]**
1–2 years		**0.67 [0.61, 0.73]**	**0.81 [0.74, 0.88]**
2–3 years		**0.70 [0.64, 0.76]**	**0.86 [0.79, 0.93]**
3–10 years		**0.78 [0.75, 0.81]**	**0.94 [0.90, 0.98]**
Time from birth of Child 3			
0–1 years		0.95 [0.84, 1.09]	**0.86 [0.75, 0.98]**
1–2 years		0.99 [0.88, 1.13]	0.91 [0.80, 1.04]
2–3 years		1.02 [0.90, 1.15]	0.96 [0.84, 1.08]
3–10 years		**1.07 [1.02, 1.13]**	**1.07 [1.02, 1.13]**
Time from birth of Child 4			
0–1 years		1.06 [0.84, 1.34]	**0.78 [0.62, 0.99]**
1–2 years		1.13 [0.90, 1.41]	0.83 [0.66, 1.04]
2–3 years		**1.73 [1.43, 2.08]**	**1.26 [1.05, 1.52]**
3–10 years		**1.50 [1.38, 1.63]**	1.09 [1.00, 1.19]
Covariates			
Year of birth	**1.03 [1.02, 1.03]**	**1.03 [1.02, 1.03]**	**1.01 [1.01, 1.01]**
Parental education			**0.89 [0.88, 0.90]**
Externalizing disorder			**4.45 [4.36, 4.55]**
Internalizing disorder			**4.70 [4.56, 4.84]**
Married			**0.83 [0.80, 0.85]**
Divorced			**1.49 [1.44, 1.56]**
FGRS_SA_			**1.25 [1.24, 1.26]**

*Note.* Model A focused on first-time parenthood and was adjusted for year of birth only. Model B further considered the birth of (up to) four children. In Model C, parental education, externalizing and internalizing disorders, marital status, and family genetic risk scores for suicide attempt were included as additional covariates. Statistically significant parameter estimates are shown in bold font. *Abbreviations:* CI, confidence interval; FGRS_SA_, family genetic risk score for suicide attempt; HR, hazard ratio.

### Time-dependent effects based on age at first birth

**Figure 1.**
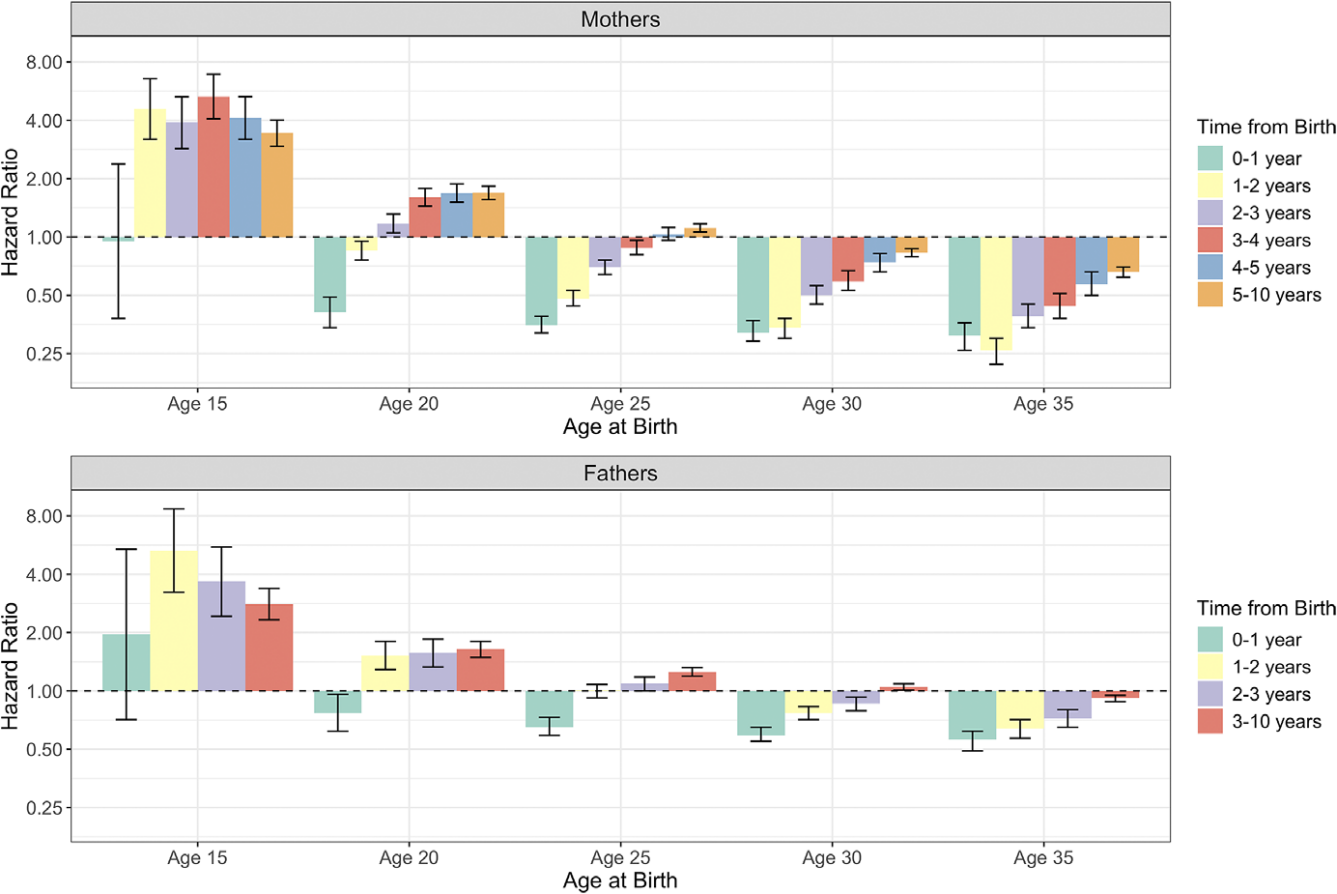
Associations between first-time parenthood and risk for suicide attempt as a function of mothers’ and fathers’ age at birth. Hazard ratios and 95% confidence intervals are shown on the y-axis. The null hypothesis (i.e. hazard ratio = 1.0) is represented by the gray dashed line. Few individuals became first-time parents at age 15, resulting in wide confidence intervals.

Next, we expanded on Models A–C above by testing whether the association between the transition to parenthood and risk for SA varies based on parental age at first birth. Statistically significant interaction effects were observed for both mothers (Supplementary Table S1) and fathers (Supplementary Table S2). To visualize these age-dependent effects, we derived separate hazard ratios for the association between parenthood and SA in 5-year increments of age at first birth (i.e. at ages 15, 20, 25, 30, and 35). Results are presented in Figure 1.

At age 15, the transition to first-time parenthood was associated with increased risk for SA in mothers (HRs = 3.43–5.30) and fathers (HRs = 2.81–5.30). The only exception was the first year following childbirth, which was not significantly related to SA risk in either parent. For older ages at first birth (ages 20 and 25 in mothers; ages 20, 25, and 30 in fathers), the initial period after childbirth was associated with reduced SA risk, whereas later time periods after childbirth were related to increased risk. This reversal in the direction of effect occurred a bit sooner after birth for fathers versus mothers. Finally, for those aged 35 years at first birth, the transition to parenthood was consistently related to decreased SA risk across the 10-year follow-up period (HRs = 0.26–0.66 for mothers, HRs = 0.56–0.92 for fathers).

### Moderation by FGRS_SA_ and parental education

Next, we evaluated whether the relationship between the transition to parenthood and risk for SA was moderated by FGRS_SA_ or parental education. Models with multiplicative interactions and age-dependent effects did not converge; therefore, we present parameter estimates from the overall models (Supplementary Tables S3 and S4). In these analyses, there was little evidence to support interactions between FGRS_SA_, parental education, and the transition to first-time parenthood. The only exception was one statistically significant interaction between FGRS_SA_ and the period 2–3 years following the birth of their first child in mothers only (HR = 1.06), which suggests that individuals with higher genetic risk benefitted more from the protective effect of parenthood.

### Considering the role of structural social support

Finally, we performed two Cox models – one model stratified by cohabitation (i.e., whether or not the parent resided with their co-parent) and one model stratified by geographic proximity to their parents – to evaluate the possibility that changes in SA risk during the first postpartum year may depend on the availability of structural social support. Parameter estimates from the overall models and models with age-dependent coefficients are presented in Table 4. For mothers, the first year following childbirth was more strongly associated with reduced SA risk if the mother resided in the same household as the father (HRs = 0.29–0.30) than if they resided separately (HRs = 0.50–0.61). A slightly different pattern of results was observed in fathers. Specifically, the first year following childbirth was associated with *lower* risk for SA if the father resided in the same household as the mother (HRs = 0.51–0.52) and with *elevated* risk for SA if they lived separately, especially for younger ages at birth (HRs = 1.25–5.48).

**Table 4.** Risk for suicide attempt during the first postpartum year as a function of cohabitation and proximity to grandparents

	Mothers HR [95% CI]					
	Overall	Age 15	Age 20	Age 25	Age 30	Age 35
Resides with father						
Yes	**0.29** **[0.26, 0.33]**	0.30 [0.08, 1.09]	**0.30** **[0.23, 0.38]**	**0.30** **[0.25, 0.34]**	**0.30** **[0.26, 0.34]**	**0.30** **[0.24, 0.26]**
No	**0.61** **[0.50, 0.74]**	2.02 [0.68, 6.03]	**0.72** **[0.58, 0.91]**	**0.60** **[0.50, 0.74]**	**0.54** **[0.43, 0.69]**	**0.50** **[0.38, 0.67]**
Proximity to maternal grandmother						
Lives in the same SAMS	1.06 [0.79, 1.42]	1.95 [0.24, 15.9]	1.07 [0.71, 1.62]	0.97 [0.70, 1.33]	0.91 [0.61, 1.35]	0.87 [0.54, 1.41]
Lives in the same municipality	**0.31** **[0.26, 0.38]**	0.78 [0.13, 4.74]	**0.37** **[0.27, 0.50]**	**0.32** **[0.27, 0.50]**	**0.30** **[0.23, 0.39]**	**0.29** **[0.20, 0.40]**
Other	**0.35** **[0.30, 0.40]**	0.96 [0.23, 4.11]	**0.42** **[0.32, 0.57]**	**0.37** **[0.29, 0.39]**	**0.34** **[0.29, 0.39]**	**0.32** **[0.26, 0.39]**

	Fathers HR [95% CI]					
	Overall	Age 15	Age 20	Age 25	Age 30	Age 35
Resides with mother						
Yes	**0.51** **[0.46, 0.56]**	0.46 [0.12, 1.73]	**0.51** **[0.38, 0.68]**	**0.51** **[0.45, 0.59]**	**0.52** **[0.47, 0.57]**	**0.52** **[0.46, 0.59]**
No	1.16 [0.99, 1.37]	**5.48** **[1.56, 19.2]**	**1.56** **[1.20, 2.04]**	**1.25** **[1.06, 1.47]**	1.10 [0.92, 1.31]	1.00 [0.80, 1.25]
Proximity to paternal grandmother						
Lives in the same SAMS	0.94 [0.74, 1.20]	1.97 [0.24, 16.2]	1.08 [0.71, 1.63]	0.97 [0.71, 1.33]	0.91 [0.61, 1.35]	0.87 [0.53, 1.41]
Lives in the same municipality	**0.52** **[0.47, 0.63]**	0.77 [0.12, 4.69]	**0.37** **[0.27, 0.50]**	**0.33** **[0.27, 0.39]**	**0.30** **[0.23, 0.39]**	**0.29** **[0.20, 0.40]**
Other	**0.63** **[0.56, 0.70]**	0.94 [0.22, 4.03]	**0.42** **[0.32, 0.57]**	**0.37** **[0.32, 0.42]**	**0.34** **[0.29, 0.39]**	**0.32** **[0.25, 0.30]**

*Note.* Analyses focused on the first year following the birth of one’s first child. Models were controlled for year of birth, parental education, externalizing and internalizing disorders, marital status, and family genetic risk scores for suicide attempt. Statistically significant parameter estimates are shown in bold font. *Abbreviations:* CI, confidence interval; HR, hazard ratio; SAMS, small area market statistics.

When considering geographical proximity to their parents, we found that the first year after childbirth was not related to SA risk when the mother/father resided in the same DESO as the maternal/paternal grandmother, respectively. However, the first year after childbirth was related to decreased SA risk when the mother/father resided in the same municipality or further from the maternal/paternal grandmother (HRs = 0.29–0.63).

## Discussion

In the present study, we investigated the relationship between the transition to parenthood and risk for nonfatal SA in a Swedish birth cohort of more than two million individuals. By considering the roles of time since birth, the number of children, parental age at first birth, and other putative risk and protective factors, we aimed to further investigate the contexts in which parenthood is protective against suicidal behavior, as well as the contexts in which parents may be at risk.

This study extends previous research on suicide death (e.g. Qin & Mortensen, 2003; Sörberg Wallin et al., 2022) by showing that parenthood status is related to decreased risk for SA in both mothers and fathers. This association persisted after accounting for their parents’ education, internalizing and externalizing disorders, marital status, and genetic liability for SA, supporting the unique and strong impact of the parenthood experience. Similarly, the association between parenthood and SA did not vary according to levels of parental education or genetic liability, demonstrating that the potentially protective effect of parenthood extends to vulnerable populations.

Nonetheless, our results also identified key contexts in which the transition to parenthood may be related to increased SA risk. First, the direction and magnitude of the effect of parenthood varied based on the number of children and their ages. The first year following childbirth was consistently associated with decreased risk for SA, which is in line with previous evidence that the protective effect of parenthood is stronger for parents of young children (Qin & Mortensen, 2003; Sörberg Wallin et al., 2022). However, we did not replicate a linear decrease in risk according to the number of children, as initially reported by Høyer and Lund (1993) in their research on suicide death. Indeed, in this study, in parents with three or four children, we observed *elevated* risk of SA in the 5- to 10-year period after childbirth. It is possible that increased SA risk in families with multiple children may reflect higher levels of parenting stress and financial strain (Hong, Zhu, & Zhao, 2023), though these hypotheses cannot be tested in the current dataset. Discrepancies between the current findings and prior work might also be explained by cohort effects. Rates of SA in Sweden have declined in recent years (Hadlaczky, 2025), and work and family policies have changed substantively, with a particular emphasis on promoting gender equality in the balance of work and childcare (Ellingsaeter & Leira, 2006). Nonetheless, birth rates in Sweden have been consistently declined since 2009 (Rossetti, n.d.), possibly indexing changes in cultural norms about large families. Therefore, the experience of having multiple children may differ for this cohort (born 1960–1980) when compared to earlier birth cohorts.

Second, it is noteworthy that the relationship between parenthood and SA varied as a function of parental age at first birth. At age 15, parenthood was consistently associated with increased risk for SA. Similar findings have been observed for other psychiatric disorders (Aitken et al., 2016; Guo et al., 2024; Tabet, Flick, Cook, Xian, & Chang, 2016). Two possible (and non-mutually exclusive) explanations for these results are that (1) individuals with a greater propensity for psychiatric disorders and suicidal behavior are more likely to have a child at a very young age and/or (2) the experience of having a child at a young age increases one’s subsequent risk for psychiatric disorders and SA. The present study was not designed to distinguish between these possibilities, though future studies may clarify the direction of effect using complementary analytic approaches, such as the difference-in-difference model. At ages 20 and 25, the initial years after childbirth were associated with reduced SA risk. However, this relationship reversed direction in later years, when raising a child may become more challenging (e.g. the transition to toddlerhood) (Hong et al., 2023; Lee, Park, Kim, Cho, & Khang, 2022; McQuillan, Bates, Staples, & Deater-Deckard, 2022). Finally, in individuals aged 30–35, the transition to first-time parenthood was related to decreased risk for non-fatal SA across 10 years of follow-up. This observation extends what has been shown in the depression literature (Pearson et al., 2019) and aligns with the proposal that age and social role transitions operate in tandem to promote emotional maturity and a sense of responsibility (Bleidorn et al., 2013).

Third, the association between parenthood and SA risk differed as a function of structural social support. Specifically, parenthood was more strongly related to reduced SA risk when co-parents lived in the same residence. In line with leading theories of suicide (Klonsky & May, 2015; Van Orden et al., 2010), it is plausible that a sense of belonging and connectedness to a partner and child may help compensate for parenting-related stress and thereby reduce the risk of SA. In addition, we found that the potentially protective effect of parenthood on SA risk was more important when the parents resided in the same municipality or further from a potential source of social support (i.e. the child’s grandmother), whereas parenthood was unrelated to SA for individuals living in the same DESO as their parents. Additional data are necessary to explore this unexpected finding, but we can reflect on three speculative and non-mutually exclusive explanations: (i) living very close to one’s parents may be a source of unmeasured relationship conflict; (ii) this effect could be driven by some individuals who live in the same residence as their parents, which may capture individuals with fewer socioeconomic resources; and/or (iii) in families where the grandmother is highly involved in the child’s care and education, it is possible that parents may feel less parental responsibility, which is an important protective factor for suicidal behavior (Bakhiyi, Calati, Guillaume, & Courtet, 2016).

The results of this study should be considered in light of several limitations. First, because our analyses focused on longitudinal, observational data, causal conclusions cannot be drawn. Future studies may apply methods that strengthen causal inference from observational data (e.g. a within-person design) to offer additional insight into the nature of the relationship between parenthood and SA. Second, it is plausible that multiple, unaddressed sources of (non-genetic) relatedness exist in the dataset (e.g. relationships between neighbors, coworkers, or friends; regional or provider-level diagnostic differences). Though it is challenging to resolve all aspects of relatedness using registry data, identifying and accounting for sources of shared variance (e.g. using mixed effects models) is an important consideration for future work. Third, it is important to consider that individuals in better physical and mental health are more likely to have children (Power et al., 2013) and may also be at lower risk of suicidal behavior. Fourth, parental education was included because many individuals in these models had not reached the average age for education completion (~25 years old) by the time of the child’s birth and/or the end of follow-up. As a result, one’s own level of education would be confounded with age (i.e. very young parents would all appear to have low levels of education). Accordingly, we included the parents’ level of education as a covariate, though this approach is not without limitations. Parents’ and offspring’s level of education are usually positively, but only moderately, correlated (r = 0.4; Hertz et al., 2008), and our approach does not account for intergenerational mobility.

Fifth, the present study focused on biological parenthood only. It will be important for future studies to evaluate whether a similar pattern of associations is observed across other pathways to parenthood (e.g. via adoption or surrogacy among opposite-sex and same-sex parents). Sixth, the registry data only capture SAs that came to the attention of medical personnel, which may underestimate the occurrence of SA. Seventh, though the Swedish registries are a valuable resource to improve our understanding of the association between parenthood and risk for SA, it is important to note that the nature of data does not allow us to capture nuances in potential confounders, such as economic status, cultural changes, or stress. For example, we included marital status as a covariate in our analyses but could not assess relationship quality.

Eighth, these analyses focused on the Swedish population. Parents in Sweden have access to numerous resources, including affordable childcare and paid parental leave, which may not be widely available elsewhere. As a result, the current findings might not be fully generalizable to other cultural contexts. However, we observed significant effects in a country where financial and instrumental child support is well developed, which suggests that effects may be even stronger in countries with less support provided by the welfare system. Finally, this study focused on parental characteristics to delineate the contexts in which the transition to parenthood may be related to SA risk; future studies could take a dyadic approach and also investigate how the child’s characteristics may moderate this association. Additional investigations of the mechanisms involved in the parenthood-SA relationship would also add valuable knowledge.

To conclude, our findings lend additional support for the inverse association between parenthood and risk for nonfatal SA. However, these results also demonstrate that parenthood is not uniformly protective: There are some subgroups (e.g. young parents, fathers living separately from their co-parent, families with multiple children) for whom the transition to parenthood is associated with increased risk for suicidal behavior, and clinical outreach may be warranted as a preventative measure. For example, screening for suicidal thoughts and behaviors at postnatal and pediatric appointments, particularly in young parents or families with multiple children, could be an important and easy-to-implement routine practice. Additionally, providing mental health information tailored to parents during these appointments might guide people toward the right resources (Calear et al., 2024).

## Supporting information

10.1017/S0033291726103262.sm001 Stephenson et al. supplementary material Stephenson et al. supplementary material

## Supplementary material

The supplementary material for this article can be found at http://doi.org/10.1017/S0033291726103262.
