# Supplementary material for: The association between the transition to parenthood and risk for nonfatal suicide attempt in a Swedish population-based sample
Source: Psychol Med. 2026 Jan 14;56:e18. doi: 10.1017/S0033291726103262 (PMC12885331; doi:10.1017/S0033291726103262)
Supplement: Stephenson et al. supplementary material [file S0033291726103262sup001.docx]

**Supporting Information:** The Association Between the Transition to Parenthood and Risk for Non-Fatal Suicide Attempt in a Swedish Population-Based Sample

Correspondence to: [Mallory.Stephenson@vcuhealth.org](mailto:Mallory.Stephenson@vcuhealth.org)

**Supplementary Methods**

**Registry Descriptions**

Multi-Generation Register. See: <https://www.scb.se/en/finding-statistics/statistics-by-subject-area/other/other/other-publications-non-statistical/pong/publications/multi-generation-register-2016/>

Register of the total population. See: <https://www.scb.se/contentassets/8f66bcf5abc34d0b98afa4fcbfc0e060/rtb-bar-2016-eng.pdf>

*National Patient Register*

In the 1960s, the National Board of Health and Welfare started to collect information regarding inpatients at public hospitals, the National Patient Register (NPR). Initially, it contained information about all patients treated in psychiatric care and approximately 16 percent of patients in somatic care. The register at that time covered six of the 26 county councils in Sweden. In 1984, the Ministry of Health and Welfare together with the Federation of County Councils decided a mandatory participation for all county councils. From 1987, NPR includes all inpatient care in Sweden. Since 2001, the register also covers outpatient doctor visits including day surgery and psychiatric care from both private and public caregivers. For more information, see https://www.socialstyrelsen.se/en/statistics-and-data/registers/register-information/the-national-patient-register/

*Primary Care Data*

We also used information from Primary Care. This is a research dataset including individual-level information on clinical diagnoses from primary health care centers. At the end of the follow-up period, the register covers almost 100% of the population. The figure below shows the percentage of the entire Swedish population that resides in counties with primary care data. For more information see: Sundquist, J., Ohlsson, H., Sundquist, K., Kendler, KS. Common adult psychiatric disorders in Swedish primary care where most mental health patients are treated. BMC Psychiatry 17, 235 (2017). https://doi.org/10.1186/s12888-017-1381-4

*
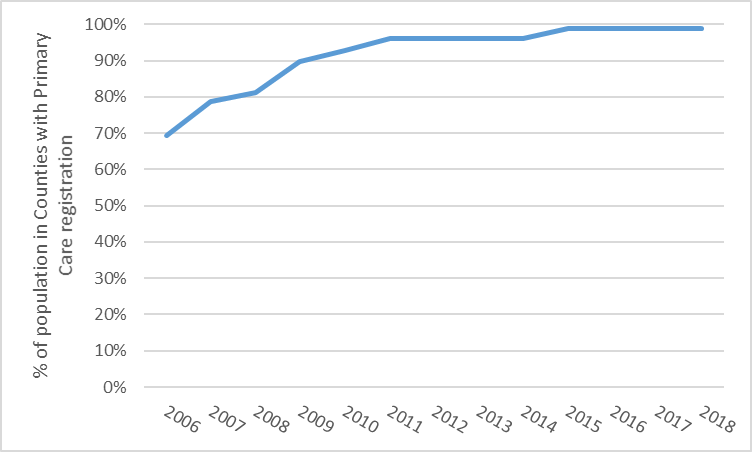
*

**Measurement of Covariates**

| Covariate | Registers Used | Definition |
| --- | --- | --- |
| Parental education | Register of the total population | Education was measured on a seven-point ordered categorical scale. We recoded these categories as years of education (7 years, 9 years, 11 years, 12 years, 14 years, 17 years, or 20 years) and took the mean across parents. |
| Externalizing | National Patient Register and Primary Care Registry and the Crime Register and Suspicion Register | Alcohol Use Disorder (AUD) was identified in the Swedish medical and mortality registries by ICD codes: ICD9: V79B, 305A, 357F, 571A-D, 425F, 535D, 291, 303, 980; ICD 10: E244, G312, G621, G721, I426, K292, K70, K852, K860, O354, T51, F10); in the Swedish Criminal Register and the Swedish Suspicion Register with at least two registrations of drunk driving (suspicion code 3005, law 1951:649 (paragraph 4 and 4A)) or drunk in charge of a maritime vessel (suspicion code 3201, law 1994:1009 (chapter 20, paragraph 4 and 5)); in the Prescribed Drug Register by the drugs disulfiram (Anatomical Therapeutic Chemical (ATC) Classification System N07BB01), acamprosate (N07BB03), and naltrexone (N07BB04).  Drug Use Disorder (DUD) was identified in the Swedish medical and mortality registries by ICD codes (ICD8: Drug dependence (304); ICD9: Drug psychoses (292) and Drug dependence (304); ICD10: Mental and behavioral disorders due to psychoactive substance use (F10-F19), except those due to alcohol (F10) or tobacco (F17)); in the Suspicion Register by codes 3070, 5010, 5011, and 5012, which reflect crimes related to drug abuse; and in the Crime Register by references to laws covering narcotics (law 1968:64, paragraph 1, point 6) and drug-related driving offences (law 1951:649, paragraph 4, subsection 2 and paragraph 4A, subsection 2). DUD was identified in individuals (excluding those suffering from cancer) in the Prescribed Drug Register who had retrieved (on average) more than four defined daily doses a day for 12 months of either Hypnotics and Sedatives (ATC Classification System N05C and N05BA) or Opioids (ATC Classification System N02A).  Criminal behavior (CB) was identified by registration in the Swedish Crime (or conviction) register, which excludes convictions for minor crimes like traffic infractions.  Externalizing behavior was a combination of AUD, DUD, and CB. |
| Internalizing | National Patient Register and Primary Care Registry | Depressive disorders were identified by ICD codes (ICD-8: 296.2, 298.0, 300.4; ICD-9: 296.2, 296.4, 298.0, 300.4; ICD-10: F32, F33).  Anxiety disorders were identified by ICD codes (ICD-8: 300.0, 300.2; ICD-9: 300A, 300C; ICD-10: F40, F41).  Internalizing behavior was a combination of depressive and anxiety disorders. |
| Married | Register of the total population | Date of marriage |
| Divorced | Register of the total population | Date of divorce |

*Family Genetic Risk Score Derivation*

| The dataset for the calculations includes:  Column1 = Identification number of the proband (Born 1932-1995)  Column2 = Identification number of the relative (1st to 5th degree relatives)  Column3 = Proportion of shared additive genetic effects (0.03125 to 0.50) with the proband  Column4 = Year of Birth of relative  Column5 = Sex of relative  Column6 = Age at registration for suicide attempt  Column7 = Age at end of follow-up (2018-12-31 or age at death, or age at emigration whichever came first) |
| --- |
| **Step 1:** Using all unique relatives with a registration for suicide attempt, we non-parametrically estimated the distribution of *Age at first registration*. The empirical distribution is used to obtain weights for relatives without a suicide attempt registration, in order to account for the proportion of the time-at-risk period they had completed at the end of follow-up. For example, for relatives at age x at end of follow-up, the weight corresponds to the proportion of relatives registered for suicide attempt that had been registered at age x. For relatives born prior to 1958, we subtracted age at the end of follow-up with the following formula: 1958 - Year of birth of relative. This modification was done in order to control for registration effects (i.e., most registers in Sweden start in 1973, suggesting that relatives from early birth cohorts do not have the possibility to be registered at younger ages). Note that all relatives with a suicide attempt are weighted one. |
| **Step 2:** Transform the binary variable (yes/no suicide attempt) into a z-score based on the threshold for the trait. The underlying liability of the individual is not assessable. Instead, we estimated the mean of the underlying liability to obtain sex and birth decade specific z-scores for relatives with a suicide attempt and relatives without a suicide attempt. We generate n random numbers from a N (0, 1) distribution and estimate the mean for relatives registered with a suicide attempt (i.e., mean of the observations above the threshold) and for relatives without a registration (i.e., mean of all observation below the threshold). The thresholds are calculated for each decade of birth and sex. |
| **Step 3**: Correct for cohabitation effects. To estimate the cohabitation effect (i.e., “shared environment”), we created a database with all individuals in the Swedish population born in Sweden 1955-1990. We also included the number of years, during ages 0-15, that individuals resided in the same household as their biological father. We thereby were able to define two kinds of families: i) “not-lived-with” father families (offspring never resided for more than 1 year in the same household or in the same community as their biological father); ii) “lived-with” father (offspring resided a minimum of 13 years in the same household as their biological father. We performed a logistic regression model with the binary trait in offspring as outcome and the binary trait in father, type of father, and their interaction as predictors. We used the interaction term as the difference of effect between genes only and genes + environment. The same approach was performed for half-siblings where we compared those who were reared together versus reared apart. The following interaction terms were used in the calculations:   \|  \| Parent/Children \| Siblings \| \| --- \| --- \| --- \| \| SA \| .66 \| .76 \| |
| **Step 4:** Calculate the product for each relative using the four components:   1. Z-score (reflecting sex and year of birth adjusted rates) 2. Weight (reflecting the proportion of risk period they had completed) 3. Cohabitation effects 4. Proportion of shared genetic effects (0.03125 – 0.5) with the proband |
| **Step 5:** Average the product calculated in step 4 across all relatives to a proband |
| **Step 6**: Correct for the number of relatives. We multiplied the results from step 5 with a shrinkage factor. Shrinkage factor (SF): B/(B+A/C). It produces more shrinkage if B and C are small and A is large.   1. the variance of the z-score across all relatives, 2. the variance in the mean z-score across all probands, 3. the weighted number of relatives for each proband (sum of Column 3 across each proband). |
| **Step 7:** Correct for differences by year of birth and county differences. There are 21 counties in Sweden. For each proband, we used the county they had resided in during the maximum number of years (measured from 1969 and onwards) We standardized the risk score by year of birth and county of the proband into a z-score with mean 0 and SD 1. This was then used as the FGRS in the analyses. |

Table S1. Assessing moderation of the association between parenthood and risk for suicide attempt by age at first birth in mothers.

|  |  | HR [95% CI] | | |
| --- | --- | --- | --- | --- |
|  |  | Model A | Model B | Model C |
| Time from birth of Child 1 | |  |  |  |
|  | 0-1 years (T1) | **1.54 [1.02, 2.35]** | **1.61 [1.07, 2.44]** | **0.58 [0.36, 0.93]** |
|  | 1-2 years (T2) | **11.8 [8.92, 15.5]** | **12.6 [9.63, 16.5]** | **4.58 [3.19, 6.56]** |
|  | 2-3 years (T3) | **30.0 [20.2, 44.5]** | **34.4 [23.6, 50.1]** | **7.55 [4.94, 11.5]** |
|  | 3-4 years (T4) | **114 [73.0, 180]** | **133 [87.0, 204]** | **20.3 [12.8, 32.2]** |
|  | 4-5 years (T5) | **144 [84.0, 244]** | **195 [118, 322]** | **18.9 [11.2, 31.9]** |
|  | 5-10 years (T6) | **189 [134, 268]** | **384 [273, 541]** | **17.9 [12.6, 25.3]** |
| Interaction with time from birth | |  |  |  |
|  | Log(age) x T1 | **0.54 [0.45, 0.64]** | **0.52 [0.44, 0.62]** | **0.81 [0.66, 0.99]** |
|  | Log(age) x T2 | **0.26 [0.23, 0.29]** | **0.26 [0.23, 0.29]** | **0.39 [0.33, 0.46]** |
|  | Log(age) x T3 | **0.21 [0.17, 0.24]** | **0.21 [0.18, 0.25]** | **0.38 [0.32, 0.46]** |
|  | Log(age) x T4 | **0.14 [0.11, 0.16]** | **0.15 [0.12, 0.18]** | **0.30 [0.25, 0.36]** |
|  | Log(age) x T5 | **0.14 [0.11, 0.17]** | **0.15 [0.12, 0.18]** | **0.33 [0.27, 0.41]** |
|  | Log(age) x T6 | **0.15 [0.13, 0.16]** | **0.13 [0.11, 0.14]** | **0.36 [0.32, 0.41]** |
| Time from birth of Child 2 | |  |  |  |
|  | 0-1 years |  | **0.34 [0.30, 0.39]** | **0.39 [0.35, 0.44]** |
|  | 1-2 years |  | **0.52 [0.47, 0.58]** | **0.64 [0.58, 0.70]** |
|  | 2-3 years |  | **0.58 [0.53, 0.64]** | **0.74 [0.67, 0.81]** |
|  | 3-4 years |  | **0.57 [0.52, 0.63]** | **0.74 [0.67, 0.81]** |
|  | 4-5 years |  | **0.68 [0.62, 0.75]** | **0.84 [0.77, 0.92]** |
|  | 5-10 years |  | **0.76 [0.72, 0.79]** | **0.90 [0.86, 0.94]** |
| Time from birth of Child 3 | |  |  |  |
|  | 0-1 years |  | **0.56 [0.47, 0.65]** | **0.51 [0.43, 0.60]** |
|  | 1-2 years |  | **0.72 [0.63, 0.83]** | **0.67 [0.58, 0.78]** |
|  | 2-3 years |  | 0.93 [0.82, 1.06] | 0.88 [0.77, 1.00] |
|  | 3-4 years |  | 1.03 [0.92, 1.17] | 0.98 [0.87, 1.10] |
|  | 4-5 years |  | **1.24 [1.11, 1.38]** | **1.18 [1.06, 1.32]** |
|  | 5-10 years |  | 0.99 [0.94, 1.05] | 1.01 [0.95, 1.07] |
| Time from birth of Child 4 | |  |  |  |
|  | 0-1 years |  | 0.83 [0.64, 1.07] | **0.62 [0.48, 0.80]** |
|  | 1-2 years |  | 1.06 [0.84, 1.32] | **0.78 [0.63, 0.98]** |
|  | 2-3 years |  | 1.23 [1.00, 1.51] | 0.90 [0.73, 1.10] |
|  | 3-4 years |  | **1.49 [1.23, 1.80]** | 1.05 [0.87, 1.27] |
|  | 4-5 years |  | **1.59 [1.32, 1.91]** | 1.11 [0.92, 1.33] |
|  | 5-10 years |  | **1.64 [1.50, 1.79]** | **1.10 [1.01, 1.20]** |
| Covariates | |  |  |  |
|  | Year of birth | **1.02 [1.02, 1.03]** | **1.02 [1.02, 1.03]** | **0.99 [0.99, 0.99]** |
|  | Parental education |  |  | **0.92 [0.91, 0.94]** |
|  | Externalizing disorder |  |  | **5.70 [5.57, 5.84]** |
|  | Internalizing disorder |  |  | **6.44 [6.23, 6.66]** |
|  | Married |  |  | **0.89 [0.86, 0.92]** |
|  |  | HR [95% CI] | | |
|  |  | Model A | Model B | Model C |
|  | Divorced |  |  | **1.62 [1.56, 1.69]** |
|  | FGRS_SA_ |  |  | **1.26 [1.25, 1.27]** |

*Note.* Model A focused on first-time parenthood and was adjusted for year of birth only. Model B further considered the birth of (up to) four children. In Model C, parental education, externalizing and internalizing disorders, marital status, and family genetic risk scores for suicide attempt were included as additional covariates. Statistically significant parameter estimates are shown in bold font. *Abbreviations.* HR = hazard ratio; CI = confidence interval; FGRS_SA_ = family genetic risk score for suicide attempt.

Table S2. Assessing moderation of the association between parenthood and risk for suicide attempt by age at first birth in fathers.

|  |  | HR [95% CI] | | |
| --- | --- | --- | --- | --- |
|  |  | Model A | Model B | Model C |
| Time from birth of Child 1 | |  |  |  |
|  | 0-1 years (T1) | **4.72 [2.75, 8.10]** | **4.95 [2.90, 8.46]** | 1.13 [0.66, 1.95] |
|  | 1-2 years (T2) | **27.9 [17.2, 45.2]** | **30.4 [18.9, 48.9]** | **5.30 [3.23, 8.70]** |
|  | 2-3 years (T3) | **39.8 [22.4, 70.7]** | **47.1 [27.0, 82.2]** | **5.87 [3.39, 10.2]** |
|  | 3-10 years (T4) | **47.7 [35.2, 64.8]** | **70.9 [52.5, 95.8]** | **5.13 [3.83, 6.89]** |
| Interaction with time from birth | |  |  |  |
|  | Log(age) x T1 | **0.45 [0.37, 0.56]** | **0.44 [0.36, 0.54]** | **0.79 [0.64, 0.97]** |
|  | Log(age) x T2 | **0.26 [0.22, 0.32]** | **0.26 [0.21, 0.31]** | **0.50 [0.41, 0.60]** |
|  | Log(age) x T3 | **0.24 [0.19, 0.30]** | **0.24 [0.19, 0.29]** | **0.51 [0.42, 0.62]** |
|  | Log(age) x T4 | **0.26 [0.23, 0.28]** | **0.24 [0.22, 0.27]** | **0.58 [0.52, 0.64]** |
| Time from birth of Child 2 | |  |  |  |
|  | 0-1 years |  | **0.55 [0.50, 0.61]** | **0.67 [0.61, 0.74]** |
|  | 1-2 years |  | **0.60 [0.55, 0.65]** | **0.77 [0.71, 0.84]** |
|  | 2-3 years |  | **0.65 [0.60, 0.71]** | **0.83 [0.76, 0.91]** |
|  | 3-10 years |  | **0.79 [0.76, 0.82]** | **0.94 [0.91, 0.98]** |
| Time from birth of Child 3 | |  |  |  |
|  | 0-1 years |  | 0.89 [0.78, 1.02] | **0.83 [0.73, 0.95]** |
|  | 1-2 years |  | 0.94 [0.83, 1.07] | 0.89 [0.79, 1.01] |
|  | 2-3 years |  | 0.98 [0.86, 1.11] | 0.94 [0.83, 1.07] |
|  | 3-10 years |  | 1.02 [0.97, 1.08] | 1.05 [1.00, 1.11] |
| Time from birth of Child 4 | |  |  |  |
|  | 0-1 years |  | 1.02 [0.81, 1.29] | **0.78 [0.61, 0.98]** |
|  | 1-2 years |  | 1.09 [0.87, 1.37] | 0.82 [0.66, 1.03] |
|  | 2-3 years |  | **1.68 [1.40, 2.02]** | **1.26 [1.04, 1.51]** |
|  | 3-10 years |  | **1.44 [1.32, 1.56]** | 1.08 [0.99, 1.17] |
| Covariates | |  |  |  |
|  | Year of birth | **1.03 [1.03, 1.03]** | **1.03 [1.03, 1.03]** | **1.01 [1.01, 1.01]** |
|  | Parental education |  |  | **0.89 [0.88, 0.90]** |
|  | Externalizing disorder |  |  | **4.41 [4.32, 4.50]** |
|  | Internalizing disorder |  |  | **4.68 [4.55, 4.82]** |
|  | Married |  |  | **0.83 [0.80, 0.85]** |
|  | Divorced |  |  | **1.48 [1.43, 1.54]** |
|  | FGRS_SA_ |  |  | **1.25 [1.24, 1.25]** |

*Note.* Model A focused on first-time parenthood and was adjusted for year of birth only. Model B further considered the birth of (up to) four children. In Model C, parental education, externalizing and internalizing disorders, marital status, and family genetic risk scores for suicide attempt were included as additional covariates. Statistically significant parameter estimates are shown in bold font. *Abbreviations.* HR = hazard ratio; CI = confidence interval; FGRS_SA_ = family genetic risk score for suicide attempt.

Table S3. Exploring potential moderators of the association between first-time parenthood and risk for suicide attempt in mothers.

|  |  | **Moderator**  HR [95% CI] | |
| --- | --- | --- | --- |
|  | | FGRS_SA_ | Education |
| Time from birth of Child 1 | |  |  |
|  | 0-1 years (T1) | **0.34 [0.31, 0.38]** | **0.34 [0.31, 0.38]** |
|  | 1-2 years (T2) | **0.47 [0.43, 0.52]** | **0.48 [0.43, 0.52]** |
|  | 2-3 years (T3) | **0.67 [0.61, 0.73]** | **0.69 [0.63, 0.75]** |
|  | 3-4 years (T4) | **0.85 [0.79, 0.93]** | **0.87 [0.80, 0.95]** |
|  | 4-5 years (T5) | 0.99 [0.91, 1.08] | 0.98 [0.90, 1.07] |
|  | 5-10 years (T6) | 0.96 [0.92, 1.01] | 0.96 [0.92, 1.00] |
| Interaction terms | |  |  |
|  | Moderator x T1 | 0.98 [0.91, 1.05] | 1.02 [0.90, 1.15] |
|  | Moderator x T2 | 1.04 [0.99, 1.09] | 0.98 [0.87, 1.10] |
|  | Moderator x T3 | **1.06 [1.01, 1.10]** | 1.00 [0.90, 1.11] |
|  | Moderator x T4 | 1.04 [1.00, 1.09] | 0.98 [0.89, 1.08] |
|  | Moderator x T5 | 1.00 [0.96, 1.04] | 0.95 [0.87, 1.04] |
|  | Moderator x T6 | 1.00 [0.98, 1.02] | 0.98 [0.94, 1.02] |
| Time from birth of Child 2 | |  |  |
|  | 0-1 years | **0.41 [0.36, 0.46]** | **0.41 [0.36, 0.46]** |
|  | 1-2 years | **0.66 [0.60, 0.73]** | **0.66 [0.60, 0.73]** |
|  | 2-3 years | **0.78 [0.71, 0.85]** | **0.78 [0.71, 0.85]** |
|  | 3-4 years | **0.78 [0.71, 0.86]** | **0.78 [0.72, 0.86]** |
|  | 4-5 years | **0.87 [0.80, 0.95]** | **0.87 [0.80, 0.95]** |
|  | 5-10 years | **0.91 [0.87, 0.95]** | **0.91 [0.87, 0.95]** |
| Time from birth of Child 3 | |  |  |
|  | 0-1 years | **0.54 [0.46, 0.64]** | **0.54 [0.46, 0.64]** |
|  | 1-2 years | **0.71 [0.62, 0.82]** | **0.71 [0.62, 0.82]** |
|  | 2-3 years | 0.92 [0.81, 1.05] | 0.92 [0.81, 1.05] |
|  | 3-4 years | 1.02 [0.91, 1.15] | 1.02 [0.90, 1.15] |
|  | 4-5 years | **1.22 [1.10, 1.37]** | **1.22 [1.10, 1.37]** |
|  | 5-10 years | 1.05 [0.99, 1.11] | 1.05 [0.99, 1.11] |
| Time from birth of Child 4 | |  |  |
|  | 0-1 years | **0.64 [0.49, 0.82]** | **0.64 [0.49, 0.82]** |
|  | 1-2 years | 0.80 [0.64, 1.00] | 0.80 [0.64, 1.00] |
|  | 2-3 years | 0.91 [0.74, 1.12] | 0.91 [0.74, 1.12] |
|  | 3-4 years | 1.07 [0.88, 1.29] | 1.07 [0.88, 1.29] |
|  | 4-5 years | 1.12 [0.93, 1.35] | 1.12 [0.93, 1.35] |
|  | 5-10 years | **1.13 [1.04, 1.24]** | **1.13 [1.04, 1.24]** |
| Covariates | |  |  |
|  | Year of birth | **0.99 [0.99, 0.99]** | **0.99 [0.99, 0.99]** |
|  | Parental education | **0.91 [0.90, 0.92]** | **0.91 [0.90, 0.92]** |
|  | Externalizing disorder | **5.84 [5.70, 5.98]** | **5.84 [5.70, 5.98]** |
|  | Internalizing disorder | **6.49 [6.28, 6.71]** | **6.49 [6.28, 6.71]** |
|  | Married | **0.90 [0.87, 0.93]** | **0.90 [0.87, 0.93]** |
|  |  | **Moderator**  HR [95% CI] | |
|  |  | FGRS_SA_ | Education |
|  | Divorced | **1.65 [1.59, 1.72]** | **1.65 [1.59, 1.72]** |
|  | FGRS_SA_ | **1.27 [1.26, 1.28]** | **1.27 [1.26, 1.28]** |

*Note.* Statistically significant parameter estimates are shown in bold font. *Abbreviations.* HR = hazard ratio; CI = confidence interval; FGRS_SA_ = family genetic risk score for suicide attempt.

Table S4. Exploring potential moderators of the association between first-time parenthood and risk for suicide attempt in fathers.

|  |  | **Moderator**  HR [95% CI] | |
| --- | --- | --- | --- |
|  | | FGRS_SA_ | Education |
| Time from birth of Child 1 | |  |  |
|  | 0-1 years (T1) | **0.60 [0.55, 0.65]** | **0.60 [0.55, 0.65]** |
|  | 1-2 years (T2) | **0.81 [0.75, 0.87]** | **0.81 [0.75, 0.88]** |
|  | 2-3 years (T3) | **0.90 [0.84, 0.97]** | **0.90 [0.84, 0.97]** |
|  | 3-10 years (T4) | 1.00 [0.97, 1.04] | 1.01 [0.97, 1.04] |
| Interaction terms | |  |  |
|  | Moderator x T1 | 1.01 [0.96, 1.06] | 1.01 [0.91, 1.12] |
|  | Moderator x T2 | 1.03 [0.98, 1.07] | 0.96 [0.88, 1.05] |
|  | Moderator x T3 | 1.01 [0.96, 1.05] | 1.00 [0.91, 1.09] |
|  | Moderator x T4 | 1.02 [1.00, 1.04] | 0.99 [0.95, 1.02] |
| Time from birth of Child 2 | |  |  |
|  | 0-1 years | **0.70 [0.64, 0.77]** | **0.70 [0.64, 0.77]** |
|  | 1-2 years | **0.81 [0.74, 0.88]** | **0.81 [0.74, 0.88]** |
|  | 2-3 years | **0.86 [0.79, 0.93]** | **0.86 [0.79, 0.93]** |
|  | 3-10 years | **0.94 [0.90, 0.98]** | **0.94 [0.90, 0.98]** |
| Time from birth of Child 3 | |  |  |
|  | 0-1 years | **0.86 [0.75, 0.97]** | **0.86 [0.75, 0.97]** |
|  | 1-2 years | 0.91 [0.80, 1.04] | 0.91 [0.80, 1.04] |
|  | 2-3 years | 0.96 [0.84, 1.08] | 0.96 [0.84, 1.08] |
|  | 3-10 years | **1.07 [1.02, 1.13]** | **1.07 [1.02, 1.13]** |
| Time from birth of Child 4 | |  |  |
|  | 0-1 years | **0.78 [0.62, 0.99]** | **0.78 [0.62, 0.99]** |
|  | 1-2 years | 0.83 [0.66, 1.04] | 0.83 [0.66, 1.04] |
|  | 2-3 years | **1.26 [1.05, 1.52]** | **1.27 [1.05, 1.52]** |
|  | 3-10 years | **1.09 [1.00, 1.19]** | **1.09 [1.00, 1.19]** |
| Covariates | |  |  |
|  | Year of birth | **1.01 [1.01, 1.01]** | **1.01 [1.01, 1.01]** |
|  | Parental education | **0.89 [0.88, 0.90]** | **0.89 [0.88, 0.90]** |
|  | Externalizing disorder | **4.45 [4.36, 4.55]** | **4.45 [4.36, 4.55]** |
|  | Internalizing disorder | **4.70 [4.56, 4.84]** | **4.70 [4.56, 4.84]** |
|  | Married | **0.83 [0.81, 0.85]** | **0.83 [0.81, 0.85]** |
|  | Divorced | **1.49 [1.44, 1.55]** | **1.49 [1.44, 1.55]** |
|  | FGRS_SA_ | **1.25 [1.24, 1.26]** | **1.25 [1.24, 1.26]** |

*Note.* Statistically significant parameter estimates are shown in bold font. *Abbreviations.* HR = hazard ratio; CI = confidence interval; FGRS_SA_ = family genetic risk score for suicide attempt.
